# A Novel Regulated Hybrid Promoter That Permits Autoinduction of Heterologous Protein Expression in Kluyveromyces lactis

**DOI:** 10.1128/AEM.00542-19

**Published:** 2019-07-01

**Authors:** Hassan Sakhtah, Juliane Behler, Alana Ali-Reynolds, Thomas B. Causey, Saulius Vainauskas, Christopher H. Taron

**Affiliations:** a New England Biolabs, Ipswich, Massachusetts, USA; b Genetics & Experimental Bioinformatics, Institute of Biology III, University of Freiburg, Freiburg, Germany; University of Illinois at Urbana-Champaign

**Keywords:** *Kluyveromyces lactis*, glucose repression, promoter, regulation, yeast

## Abstract

The yeast Kluyveromyces lactis has been a successful host for the production of heterologous proteins for over 30 years. Currently, the galactose-/lactose-inducible and glucose-repressible *LAC4* promoter (P*_LAC4_*) is the most widely used promoter to drive recombinant protein expression in K. lactis. However, P*_LAC4_* is not fully repressed in the presence of glucose and significant protein expression still occurs. Thus, P*_LAC4_* is not suitable in processes where tight regulation of heterologous gene expression is required. In this study, we devised a novel K. lactis promoter system that is both strong and tightly controllable. We first tested several different endogenous K. lactis promoters for their ability to express recombinant proteins. A novel hybrid promoter (termed P_350_) was created by combining segments of two K. lactis promoters, namely, the strong constitutive P*_GAP1_* promoter and the carbon source-sensitive P*_ICL1_* promoter. We demonstrate that P_350_ is tightly repressed in the presence of glucose or glycerol and becomes derepressed upon depletion of these compounds by the growing cells. We further illustrate the utility of P_350_-controlled protein expression in shake flask and high-cell-density bioreactor cultivation strategies. The P_350_ hybrid promoter is a strong derepressible promoter for use in autoinduction of one-step fermentation processes for the production of heterologous proteins in K. lactis.

**IMPORTANCE** The yeast Kluyveromyces lactis is an important host for the expression of recombinant proteins at both laboratory and industrial scales. However, the system lacks a tightly regulated promoter that permits controlled expression of heterologous proteins. In this study, we report the engineering of a highly regulated strong hybrid promoter (termed P_350_) for use in K. lactis. P_350_ is tightly repressed by glucose or glycerol in the medium but strongly promotes gene expression once the carbon source has been consumed by the cells. This feature permits heterologous protein expression to be “autoinduced” at any scale without the addition of a gratuitous inducer molecule or changing feed solutions.

## Abstract (precis)

The yeast Kluyveromyces lactis is an important host for the expression of recombinant proteins at both laboratory and industrial scales. However, the system lacks a tightly regulated promoter that permits controlled expression of heterologous proteins. In this study, we report the engineering of a highly regulated strong hybrid promoter (termed P_350_) for use in K. lactis. P_350_ is tightly repressed by glucose or glycerol in the medium but strongly promotes gene expression once the carbon source has been consumed by the cells. This feature permits heterologous protein expression to be “autoinduced” at any scale without the addition of a gratuitous inducer molecule or changing feed solutions.

## INTRODUCTION

For over 30 years, the yeast Kluyveromyces lactis has been used as a host for the expression of heterologous proteins (1, 2). K. lactis has many attributes that make it suitable for protein expression; it is easily genetically manipulated, has a sequenced genome (3, 4), has a Crabtree-negative metabolism (5, 6), and can be cultivated using simple growth media. Over 100 heterologous proteins have been expressed in K. lactis (1, 2). Additionally, recombinant K. lactis expression has been performed at an industrial scale to produce recombinant prochymosin for use in cheese manufacturing (7). As such, K. lactis has a long history of safe use in the food industry and is a generally regarded as safe (GRAS) microorganism (8). Combined, these features have made K. lactis an attractive and useful yeast expression system for both laboratory and scaled processes.

Despite the long history of this organism as an expression system, only a small number of promoters have been tested for their ability to efficiently express foreign genes in K. lactis (1, 2). Furthermore, no regulated promoter that functions with the same strength and tight regulation as the methanol-inducible *AOX1* promoter (9) from Pichia pastoris has been described for this yeast. The most often used expression strategy employs the *K. lactis LAC4* promoter (P*_LAC4_*), a strong promoter that regulates endogenous β-galactosidase (Lac4p) expression in response to lactose or galactose (10–14). While P*_LAC4_* can achieve up to 100-fold induction in the presence of lactose (10), it is not tightly repressed in the absence of its inducer, leading to significant background expression of a target protein. This poses challenges for the expression of toxic or complex proteins (15–17). Additionally, serendipitous bacterial Pribnow-like sequences in P*_LAC4_* have caused detrimental leaky expression of heterologous genes during expression vector assembly in Escherichia coli (17, 18). Thus, there is still a need for a K. lactis promoter system that is strong, tightly regulated, and applicable to protein production strategies at any scale.

Both constitutive and inducible promoters have been used for protein production by K. lactis and other yeasts (1, 2, 17, 19, 20). In addition, regulated gene expression has been achieved with derepressible carbon source-controlled promoters (19, 21, 22). With these promoters, gene expression is autoinduced when the carbon source, which normally represses the promoter, is depleted from the growth medium. This scheme permits simple, scalable, and highly regulated protein production processes where biomass accumulation and protein expression steps are separated. Biomanufacturing processes using derepressible promoters have recently been gaining significant interest (19, 22).

In this study, we report the development of a novel strong and autoinducible promoter system for K. lactis gene expression. We initially surveyed a selection of endogenous K. lactis promoters for their suitability for heterologous protein expression. Portions of two promoters, the strong constitutive P*_GAP1_* promoter and the carbon source-sensitive P*_ICL1_* promoter (from the *K. lactis GAP1* and *ICL1* genes, respectively), were combined to form a novel hybrid-regulated promoter termed P_350_. We showed that P_350_ directs robust recombinant protein expression that is sharply induced by the depletion of glucose or glycerol from the culture medium upon consumption by the growing cells. We show the utility of P_350_ in both shake flask and high-density fermenter expression strategies to tightly repress protein production during biomass accumulation and then autoinduce protein expression. P_350_ is the first autoinducible promoter reported for K. lactis and is applicable to protein expression strategies at any scale.

## RESULTS

### Survey of endogenous K. lactis promoters.

**FIG 1:**
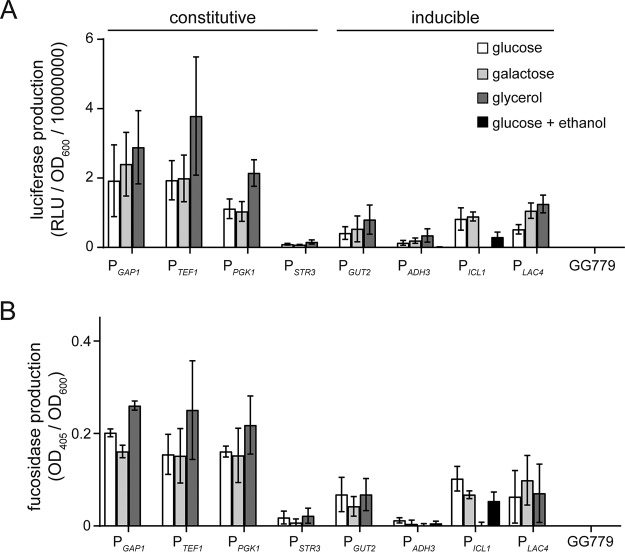
*Gaussia* luciferase (A) or α-l-fucosidase (B) expression in K. lactis in response to different promoters and carbon sources. For each strain, the average protein production from five individual cultures, each inoculated with a single transformant, is shown. Cells were grown in deep-well plates containing YEP medium supplemented with different carbon sources for 48 h at 30°C. Ethanol was added to “glucose + ethanol” cultures after 24 h. Protein production at 48 h was determined by normalizing the activity of the reporter protein (RLU for luciferase or OD_405_ for α-l-fucosidase) to the corresponding cell density (OD_600_) of each culture. Cultures of the wild-type K. lactis GG799 strain were used as negative controls. Error bars represent the standard deviation of the mean of five different cultures.

Relatively few K. lactis promoters have been explored for their ability to efficiently drive heterologous protein expression. Thus, we conducted a survey of the performance of several promoters that have been described in various biochemical and genetic studies (Table 1) (3, 14, 23–29). We measured the expression of two reporter genes (*Gaussia* luciferase and α-l-fucosidase) under the control of eight different K. lactis carbon source catabolism gene promoters. Linear expression cassettes for each promoter-reporter combination were constructed using *in vitro* DNA assembly (30) (see Fig. S1 in the supplemental material). These constructs were individually inserted into the K. lactis chromosome via integrative transformation. Protein production was assessed by growth of each strain in deep-well culture plates containing yeast extract-peptone (YEP) medium supplemented with different carbon sources. Due to significant variability in expression yields reported among individual clones within K. lactis transformant populations (31), 15 randomly chosen transformants for each expression construct were grown. The spent medium from each culture was assayed for reporter enzyme activity. Optimal expression of each promoter was determined by calculating the average activity from the five best-expressing clones (Fig. 1).

**TABLE 1:** *K. lactis* promoters tested in this study

Promoter	Expressed protein	Promoter regulation	Inducer	Regulatory region	Reference(s)
P*_GAP1_*	Glyceraldehyde-3-phosphate dehydrogenase	Constitutive	NA^*a*^	NA	23
P*_TEF1_*	Translational elongation factor EF-1α	Constitutive	NA	NA	3
P*_PGK1_*	Phosphoglycerate kinase	Constitutive	NA	NA	24
P*_STR3_*	Cystathionine beta-lyase	Constitutive	NA	NA	25
P*_ADH3_*	Alcohol dehydrogenase 3	Inducible	Glycerol	Unknown	28
P*_GUT2_*	Glycerol-3-phosphate dehydrogenase	Inducible	Glycerol	Unknown	29
P*_ICL1_*	Isocitrate lyase	Inducible	Ethanol	−811 to −690	26, 27
P*_LAC4_*	Lactase	Inducible	Galactose/lactose	−673 to −149	14

a NA, not applicable.

Several protein expression patterns were observed in this promoter comparison. First, strains harboring the most constitutive promoter constructs showed the highest overall reporter protein expression under each of the growth conditions tested (Fig. 1). The only exception was that P*_STR3_* consistently exhibited low reporter protein expression. Second, growth in medium containing glycerol generally increased reporter protein production in all constitutive promoter construct-containing strains. Third, growth in medium containing ethanol abolished reporter protein expression in almost all strains (P*_ICL1_* strains being the exception). Finally, reporter protein expression from each strain harboring an inducible promoter construct was significantly lower than that observed for constitutive promoters (Fig. 1). Thus, no single promoter yielded both of the traits (e.g., strength and tight regulation) that are desirable for optimal heterologous protein production. However, the constitutive promoters P*_GAP1_* and P*_TEF1_* both showed considerable strength, and P*_ICL1_* showed tight repression in glycerol. Therefore, we elected to further explore if highly regulated hybrid promoters that combine the strength of P*_GAP1_* (P*_TEF1_* was arbitrarily not pursued) with the tight regulation of P*_ICL1_* could be engineered.

### Creation of novel P*_ICL1_*-P*_GAP1_* hybrid promoters.

**FIG 2:**
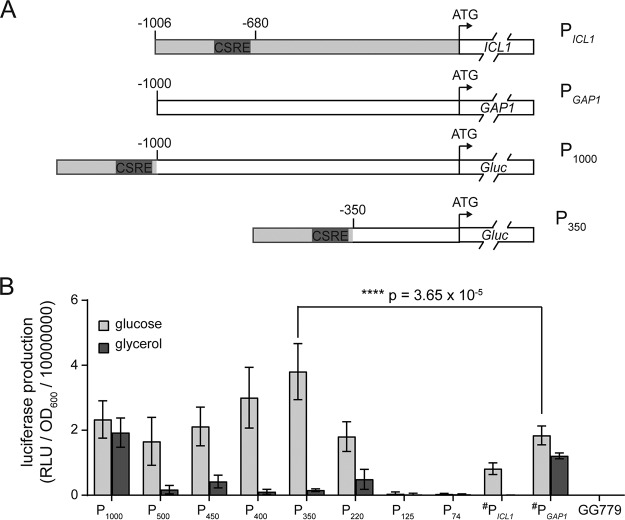
*Gaussia* luciferase expression using the P*_ICL1_*-P*_GAP1_* fusion promoters in response to different carbon sources. (A) Diagrams of promoters P*_ICL1_*, P*_GAP1_*, P_1000_, and P_350_. The region between nucleotides −690 and −811 upstream from the *ICL1* translation start site contains two carbon source-responsive elements (CSRE; shown in dark gray). A series of promoters was created by fusing the regulatory portion of P*_ICL1_* to progressively shorter regions of the P*_GAP1_* promoter relative to the *GAP1* translation start site (i.e., P_1000_, P_500_, P_450_, P_400_, P_350_, P_220_, P_125_, and P_74_). Shown in panel A, P_350_ contains 350 nucleotides immediately upstream of the *GAP1* translation start site fused to the P*_ICL1_* regulatory fragment. (B) Average protein production from 10 individual strains harboring each hybrid promoter fused to DNA encoding *Gaussia* luciferase (Gluc). Control strains where four biological replicates were analyzed are indicated by the # symbol. All cultures were grown in deep-well plates containing YEP medium supplemented with different carbon sources for 48 h at 30°C, after which Gluc activity (RLU) was normalized to the cell density (OD_600_) for each culture. Cultures of wild-type K. lactis GG799 cells were used as negative controls. Error bars represent the standard deviation from the mean.

In the K. lactis *ICL1* promoter, two previously reported carbon source-responsive elements (CSREs) that repress expression in glycerol are located between nucleotides −690 and −811 upstream of the translation start site (Fig. 2A) (27). Using *in vitro* DNA assembly, the regulatory region of P*_ICL1_* was fused to 1,000 bp of P*_GAP1_* DNA immediately upstream of the *K. lactis GAP1* translation start codon. This hybrid promoter was termed P_1000_. From P_1000_, a nested series of smaller promoters was created by fusing the same P*_ICL1_* regulatory fragment to progressively smaller regions of P*_GAP1_* (Fig. 2).

To test their strength and regulation, each hybrid promoter was fused to DNA encoding the *Gaussia* luciferase reporter protein (Gluc) and inserted into the K. lactis chromosome by integrative transformation. Expressed luciferase activity was measured in the spent medium of transformants grown in deep-well plates containing YEP supplemented with either 2% glucose or 2% glycerol. In both glucose- and glycerol-containing medium, P_1000_-Gluc strains produced slightly more luciferase activity than strains expressing Gluc from wild-type P*_GAP1_* (Fig. 2B), indicating that the largest hybrid promoter retained its ability to function as a strong promoter. However, P_1000_ did not exhibit repression in glycerol. In contrast, P_500_ through P_220_ each showed comparable or better strength than P*_GAP1_* and significant repression in glycerol. Of these, P_350_ and P_400_ showed both the highest Gluc expression in glucose and the strongest repression in glycerol. The smaller hybrid promoters P_125_ and P_74_ had no ability to produce luciferase in either carbon source. Therefore, P_350_ was selected for further comparison to P_1000_ and P_125_ in subsequent expression experiments.

### Protein expression from the hybrid P_350_ promoter.

**FIG 3:**
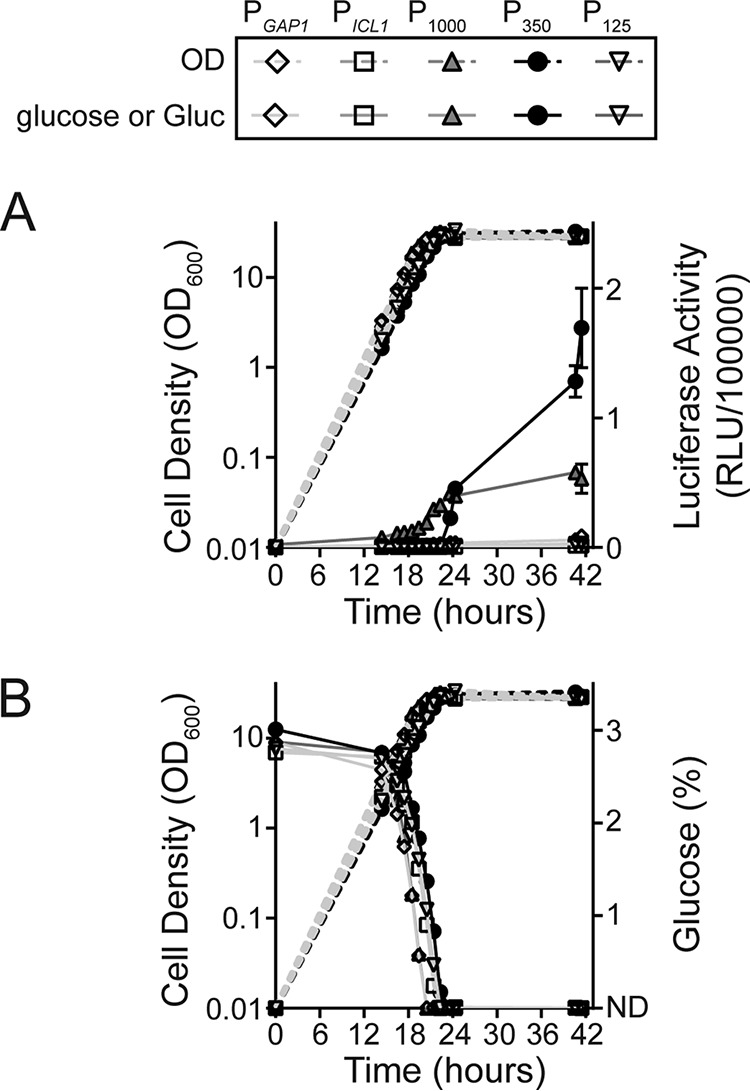
Gluc expression and glucose consumption during culturing of strains containing hybrid promoter-Gluc constructs. K. lactis GG799 cells harboring P*_GAP1_*-Gluc, P*_ICL1_*-Gluc, P_1000_-Gluc, P_350_-Gluc, and P_125_-Gluc expression constructs were grown in YDFM containing glucose in bioreactors. Shown are (A) luciferase activity (RLU/100,000) and cell growth (OD_600_) relative to hours of culturing, and (B) glucose consumption (%) and cell growth (OD_600_) relative to hours of culturing. P_1000_-Gluc showed constitutive Gluc expression higher than that of P*_ICL1_*-Gluc or P*_GAP1_*-Gluc. P_350_-Gluc showed Gluc expression only after glucose had been consumed from the culture medium. No Gluc expression was observed with P_125_-Gluc. ND represents the glucose detection limit (<0.002%) of our instrumentation. Error bars represent the standard deviation from the mean of three independent fermentations.

To better compare the P_1000_, P_350_, and P_125_ hybrid promoters, we grew the different luciferase expression strains in yeast-defined fermentation medium (YDFM) containing glucose as the sole carbon source in batch cultivation in a bioreactor. Luciferase production and nutrient consumption were monitored over time. All strains grew similarly under these conditions; however, there were marked differences in luciferase production between them (Fig. 3A). Significantly more luciferase was produced by strains using P_1000_-Gluc or P_350_-Gluc constructs than those using P_125_-Gluc or wild-type P*_GAP1_*-Gluc and P*_ICL1_*-Gluc control constructs. By 14.4 h, luciferase activity was detectable in the culture medium of the strain containing the P_1000_-Gluc construct, and luciferase production increased steadily until ∼23.7 h, after which it significantly slowed. No luciferase activity was detectable in cultures harboring the P_350_-Gluc construct until 22.3 h, after which it sharply increased to significantly higher levels than the strain with the P_1000_-Gluc construct (Fig. 3A).

Glucose consumption profiles revealed that all Gluc expression strains consumed the carbon source at similar rates (Fig. 3B). Luciferase activity was detectable in cultures of strains containing the P_1000_-Gluc construct before glucose was completely depleted around 23 h. However, no luciferase was produced by cultures containing the P_350_ construct until glucose was no longer detectable, supporting the conclusion that glucose depletion may derepress P_350_ and initiate luciferase expression.

### Carbon source regulation of P_350_ function in shake flasks.

We further explored if P_350_ could be used to robustly express heterologous proteins in response to carbon source limitation in shake flasks. Because K. lactis produces ethanol under hypoxic conditions (5, 6) and we found *Gaussia* luciferase to be sensitive to ethanol accumulation in growth medium (see Fig. S2 in the supplemental material), we used bovine enterokinase light chain (EK_L_) as a reporter enzyme. In these experiments, we utilized a novel K. lactis strain (YCT1267) in which DNA encoding EK_L_ was cloned downstream of P_350_ and integrated into the K. lactis chromosome.

**FIG 4:**
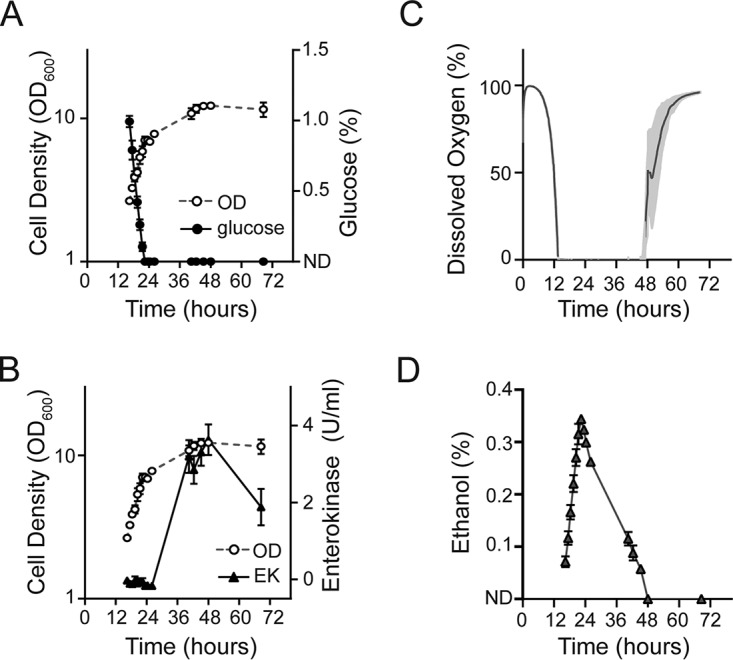
P_350_-dependent production of bovine EK_L_ by K. lactis YCT1267 cultured in shake flasks. The cultures were grown in YDFM containing 1% glucose as the sole carbon source. Shown are (A) glucose consumption, (B) EK_L_ expression, (C) oxygen consumption, and (D) ethanol production relative to hours of culturing. In panels A and D, ND represents the glucose (<0.002%) and ethanol (<0.05%) detection limits of our instrumentation. Error bars (and light gray shading in panel B) represent the standard deviation from the mean of three independent cultures.

To test if our P_350_-EK_L_ model system produced EK_L_ in response to glucose depletion, YCT1267 was grown in PreSens shake flasks (that permit culture pH and dissolved oxygen to be monitored in real-time) supplemented with 1% glucose. EK_L_ activity was detected after 40.5 h of growth. The dissolved oxygen in the culture dropped to zero percent after 12 h and ethanol was detected after 16.4 h of growth (Fig. 4C). Ethanol (which can also act as a carbon source) accumulated throughout the experiment and reached a maximum concentration of 0.35% after 22.5 h (Fig. 4D), corresponding to the time when culture medium glucose became depleted (Fig. 4A). The ethanol concentration in the culture medium did not drop below the detection limit until 48.0 h (Fig. 4D), at which time the highest level of EK_L_ activity was observed (Fig. 4B). Thus, while the native *K. lactis ICL1* promoter is typically induced in the presence of ethanol (26, 27), physiological levels of ethanol produced by the culture did not appear to strongly influence P_350_ (Fig. 4A to D) and induction of EK_L_ expression correlated most strongly to glucose depletion.

We further tested if EK_L_ production was influenced by the starting concentration of the carbon source. YCT1267 was grown in PreSens shake flasks, using medium containing 0.5%, 1%, or 2% glucose. Shake flask cultures quickly became hypoxic, and the formation of fermentation products drastically reduced the growth rate of the cultures (see Fig. S4 in the supplemental material). Ethanol production increased in inverse proportion to the concentration of glucose. Cultures consumed 0.5% glucose after 19 h, 1% glucose after 21 h, and 2% glucose after 26 h. The dissolved oxygen returned to 100% after 36 h in the culture that contained 0.5% glucose, 60 h in the culture that contained 1% glucose, and 139 h in the culture that contained 2% glucose. In general, EK_L_ production from P_350_ correlated with increasing amounts of carbon source available in each shake flask culture. However, of the starting glucose concentrations, 1% glucose gave the highest yield after extended culture growth (Fig. S4E) with minimal ethanol production (Fig. S4D).

Considered together, these shake flask experiments support the conclusion that the P_350_ hybrid promoter becomes derepressed through the depletion of glucose over the course of normal cellular growth. Furthermore, these observations suggest that the P_350_ hybrid promoter could be used to turn on (or “autoinduce”) heterologous protein expression in a bioprocessing routine with no manual intervention (e.g., addition of a gratuitous inducer or change of feed solution).

### Regulated protein production using P_350_ in bioreactor cultivation.

**FIG 5:**
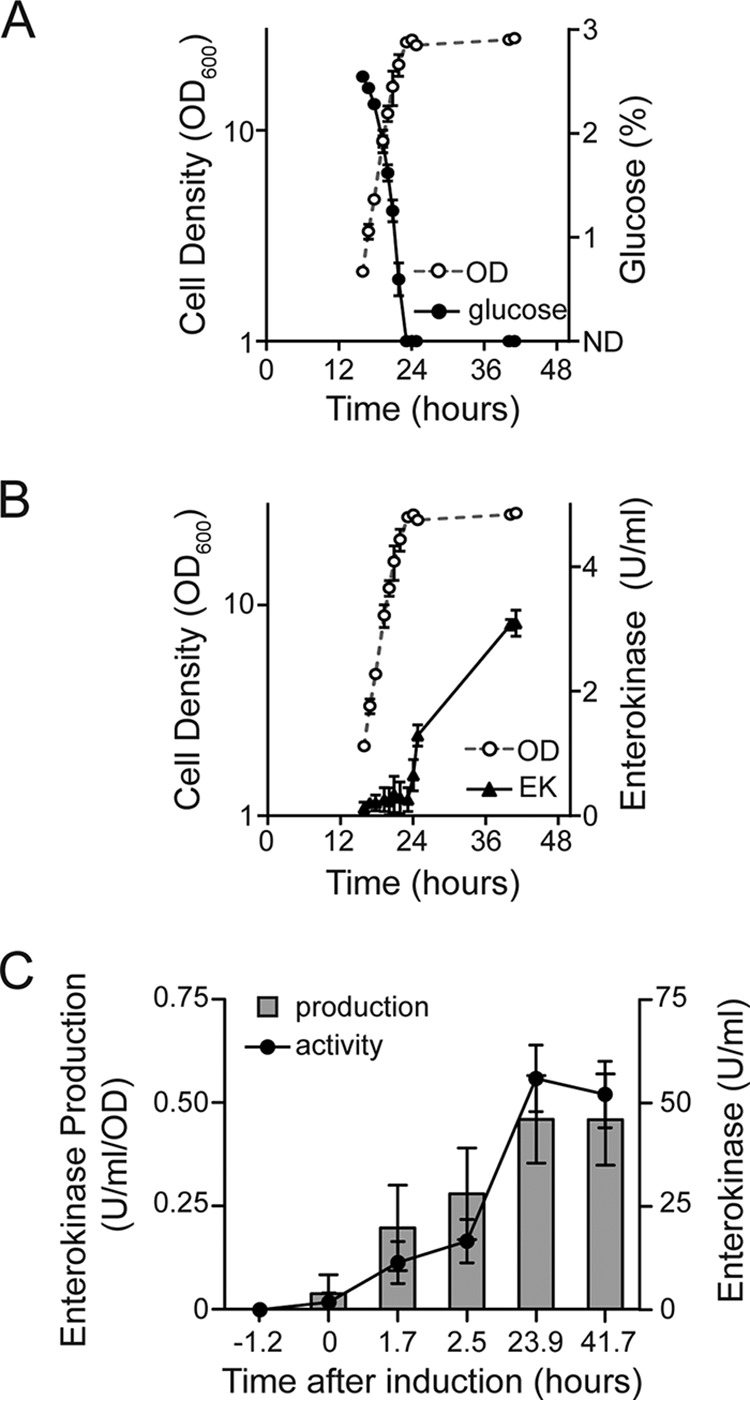
P_350_-dependent production of bovine EK_L_ by K. lactis YCT1267 cultured in bioreactors. Cultures were grown in YDFM containing 2% glucose as the sole carbon source. Shown are (A) glucose consumption and (B) EK_L_ expression. (C) High-cell-density production of EK_L_. After 42 h of fed-batch biomass accumulation, glucose feeding was stopped (0-h time point) and EK_L_ expression was induced. In panel A, ND represents the glucose detection (<0.002%) limit of our instrumentation. Error bars represent the standard deviation from the mean of three independent fermentations.

When growth of YCT1267 was performed in a bioreactor, EK_L_ production again strongly correlated with carbon source depletion. For this experiment, a batch fermentation process in which YCT1267 was grown in YDFM containing 2% glucose was performed in a bioreactor. EK_L_ activity was first detected after 24.1 h of growth, approximately 55 min after glucose was no longer detectable in the culture medium (Fig. 5A). The beginning of EK_L_ production occurred at the same time the culture stopped consuming oxygen and dissolved oxygen in the medium began to rise (see Fig. S3 in the supplemental material). Both are signatures of glucose depletion and a resulting change in cellular respiration. EK_L_ activity continued to rise throughout the remainder of the experiment, and maximum activity was achieved after 41 h (Fig. 5B). Dissolved oxygen was maintained at 30% in the culture, and ethanol was not detected. The lack of ethanol, an alternative carbon source, in the growth medium may explain the tighter correlation between glucose depletion and initiation of EK_L_ production in the bioreactor than what was observed in shake flasks.

We further examined if a high-cell-density fed-batch cultivation process could be used to scale EK_L_ production using P_350_. YCT1267 was grown in YDFM containing 2% glucose in a bioreactor. After 18 h, a 60% glucose solution was added to the culture at a constant rate (5 ml/h) from 18 to 42 h to achieve high-cell-density growth (optical density at 600 [OD_600_], 128.7). Glucose remained limiting throughout the experiment, and no ethanol was detected in the medium (see Fig. S5 in the supplemental material). After 42 h of biomass accumulation, the glucose feed was stopped, after which EK_L_ activity in the medium quickly increased to >10-fold more activity than what was observed in the batch cultures (Fig. 5C). In a separate experiment, we showed that glycerol could also be used to regulate P_350_ (see Fig. S6 in the supplemental material). In that experiment, the glycerol concentration accumulated to a maximum of 11%; however, no ethanol was detected. EK_L_ production correlated with glycerol depletion, although to slightly lower levels than in cultures grown using glucose (Fig. S6; Fig. 3B). These experiments illustrate that the pattern of autoinducible protein expression observed with P_350_ in shake flasks is more tightly controlled in fed-batch bioreactors and can be easily scaled to high-cell-density processes.

## DISCUSSION

In this study, a survey of several constitutive and inducible K. lactis carbon catabolite gene promoters was conducted to evaluate their ability to efficiently direct recombinant protein expression in K. lactis. Strong constitutive promoters and a promoter showing tight carbon source regulation (P*_ICL1_*) were observed, although no promoter exhibited both traits. As such, we engineered a hybrid promoter (P_350_) that combined the robustness of the strong constitutive *GAP1* promoter and the tight regulation of the *ICL1* promoter. We showed that tight repression of P_350_ occurred until the carbon source (glucose or glycerol) was depleted from the growth medium, after which robust heterologous protein expression was achieved. Finally, we demonstrated strategies for autoinduction of recombinant protein expression from P_350_ in both shake flasks and high-cell-density bioreactors. Our study concludes that the P_350_ hybrid promoter will permit the development of novel one-step fermentation processes for heterologous protein production in K. lactis.

For the production of nontoxic proteins, a strong constitutive expression system can help minimize cultivation efforts, leading to higher volumetric productivity (32). In this study, the three K. lactis constitutive promoters P*_GAP1_*, P*_TEF1_*, and P*_PGK1_* were shown to be effective promoters, and each had similar or stronger promoter activity than the commonly used K. lactis P*_LAC4_* (17). Thus, the combined strength and ability to use these promoters in various carbon sources makes them attractive alternatives to P*_LAC4_* for the expression of heterologous genes in K. lactis. The *GAP1* promoter has also been successfully employed in other yeasts. In P. pastoris, P*_GAP_* has been used as a constitutive alternative to the methanol-regulated P*_AOX1_* that is routinely used to produce heterologous proteins in this yeast (33). Additionally, P*_GAP_* has also been successfully used for the constitutive expression of heterologous proteins in Saccharomyces cerevisiae (34).

The inducible promoters examined in this study did not display higher expression than that observed with P*_LAC4_*. Only P*_ICL1_* showed both comparable strength to P*_LAC4_* and tight carbon source-dependent regulation. It has been reported previously that the K. lactis *ICL1* promoter is induced by ethanol and repressed by glucose and glycerol (27). In contrast to its S. cerevisiae homolog, which is only weakly repressed by glycerol when grown together with ethanol, *K. lactis ICL1* expression is strongly repressed by glycerol alone through a Cat8-independent mechanism (27). Our examination of protein expression from native P*_ICL1_* in K. lactis revealed that glycerol repression was remarkably tight throughout our tested cell cultivation period (up to 72 h), whereas in medium containing only glucose, the promoter became derepressed once glucose was consumed. Additionally, ethanol induction of the promoter did not result in high expression of recombinant protein (Fig. 1). These observations were in agreement with prior studies of P*_ICL1_* in P. pastoris and S. cerevisiae, where glucose derepression of P*_ICL1_* resulted in higher reporter gene expression than with ethanol induction (35, 36).

A set of hybrid promoters was created by combining functional elements of P*_ICL1_* and P*_GAP1_*. Several of these hybrids retained desirable functional traits of both parental promoters. The hybrid promoter P_350_ was highly active and yielded stronger gene expression than wild-type P*_GAP1_*. Additionally, it showed tight repression in the presence of glycerol or glucose, a trait associated with native P*_ICL1_*. We exploited the features of P_350_ to achieve robust expression of heterologous proteins upon depletion of a carbon source from the culture medium. Thus, P_350_ will enable novel scalable one-step fermentation strategies for recombinant protein production in K. lactis. For example, accumulation of cellular biomass can be achieved in any sized bioreactor in the presence of glucose (or glycerol) where heterologous protein expression will be tightly repressed. At a desired point, the glucose feed can be stopped, residual glucose will be rapidly consumed by the cells, and strong expression of the heterologous protein will be induced. This approach avoids the addition of a gratuitous inducer, the need to change feed solutions, and potential process variations sometimes associated with mixing in large fluid volumes. This process is also possible at small scale (e.g., shake flasks). Here, batch cultivation of cells in a fixed concentration of a carbon source will allow for cell growth until it is consumed, after which protein expression will commence.

In summary, our study advances the field of K. lactis protein expression by describing the first autoinducing promoter for use with this host organism. The novel P_350_ hybrid promoter will enable the development and implementation of one-step protein expression methods for both small- and large-scale bioprocesses.

## MATERIALS AND METHODS

### Plasmid and strain construction.

The K. lactis GG799 expression strain (17) was used throughout this study. Unless otherwise stated, K. lactis cultures used for strain construction were typically grown in YEP medium consisting of 1% (wt/vol) yeast extract and 2% (wt/vol) soytone supplemented with 2% (wt/vol) glucose or 2% (wt/vol) glycerol as a carbon source.

A diagram of the expression vector assembly strategy used in this study is presented in Fig. S1. All primers and plasmids were designed using the NEBuilder assembly tool version 2.0.8 (http://nebuilder.neb.com/) and are listed in Table S1 in the supplemental material. DNA fragments were amplified with Q5 high-fidelity DNA polymerase (New England Biolabs, Ipswich, MA) and purified using the QIAquick gel extraction kit (Qiagen, Valencia, CA). To assemble the expression vectors, 50 ng of EcoRI-digested pUC19 and 100 ng of each DNA fragment to be inserted were added to an NEBuilder HiFi DNA assembly master mix reaction (New England Biolabs). A 2-μl aliquot of the *in vitro* assembly reaction mixture was used to transform 5-alpha competent E. coli cells (New England Biolabs). Transformants were selected on LB agar plates supplemented with 100 μg/ml ampicillin. The resulting K. lactis expression vectors (referred to as pUC19/Pr) containing different promoters were used for the assembly of expression cassettes carrying a reporter gene. The *Gaussia princeps* luciferase (GLuc; EC 1.13.12.5), α-l-fucosidase (EC 3.2.1.51), and bovine enterokinase light chain (EK_L_; EC 3.4.21.9) reporter genes were amplified from plasmids containing a corresponding cloned cDNA. Reporter gene expression cassettes were assembled *in vitro* as described above.

For expression in yeast, a purified linearized expression cassette (0.1 μg) was introduced into K. lactis GG799 competent cells, as described by the manufacturer (New England Biolabs). Transformants were selected by growth on nitrogen-free yeast carbon base (YCB) agar medium (New England Biolabs) supplemented with 5 mM acetamide for 3 to 4 days at 30°C.

Strains were verified using PCR and genomic DNA sequencing. Genomic DNA was isolated using a lithium acetate-sodium dodecyl sulfate (LiOAc-SDS) recovery method (37). Briefly, the cell pellet from a 1.5-ml saturated liquid culture was suspended in 400 μl of 200 mM LiOAc-1% SDS (wt/vol) solution and incubated at 70°C for 15 min. DNA was precipitated by adding 600 μl of 96% ethanol (vol/vol) and the samples were centrifuged at 16,000 × *g* for 5 min. The DNA pellet was suspended in 30 μl distilled water and used as the template for PCR and nucleotide sequencing.

### Culture conditions for screening reporter protein activity.

A preculture of K. lactis strains was prepared by scraping and suspending a small volume of cells in 2 ml YEP medium containing 2% (wt/vol) glucose. The cells were grown for 24 h to obtain uniform densities of individual strains. For initial screening of reporter proteins under the control of different promoters, cultures of K. lactis strains were grown in 48-well deep-well plates (Axygen Scientific, CA) containing 2 ml YEP medium supplemented with different carbon sources. These cultures were inoculated with 10 μl of preculture to a cell density of ∼0.15 OD_600_ units, as measured using a Genesys spectrophotometer (Thermo Fisher Scientific, Waltham, MA). The plates were sealed with AirPore tape sheets (Qiagen) and the cultures were grown at 30°C for 48 h with shaking at 200 rpm. Cleared spent culture medium was obtained by centrifugation at 3,200 × *g* for 10 min. Samples were stored at 4°C until further analyzed.

### Seed culture preparation.

Seed cultures were used to inoculate growth media for all shake flask and fermenter experiments. To create a frozen seed culture stock, a desired K. lactis strain (e.g., GG799 or YCT1267) was streaked onto nitrogen-free yeast carbon base agar medium supplemented with 5 mM acetamide and incubated at 30°C for 48 h. Cells were then scraped from the plate and suspended in 3 ml of standard E. coli M9 minimal salts buffer. A Dasgip bioblock reactor containing 1 liter of yeast-defined fermentation medium (YDFM) (38) supplemented with 2% (wt/vol) glucose, 100 μg/ml ampicillin, and 0.01% (vol/vol) Antifoam 204 (Sigma-Aldrich, St. Louis, MO) was inoculated using the cell suspension to an initial OD_600_ of ∼0.008. The pH was maintained at 5.65 using 28% (wt/vol) ammonium hydroxide and 10% (vol/vol) phosphoric acid. The culture was grown at 30°C and initially agitated at 500 rpm. When the dissolved oxygen reached 30% air saturation, an agitation/gas flow/oxygen enrichment cascade was used to maintain this level of oxygenation. When the culture density reached 1.0 to 3.5 OD_600_ units, the culture was transferred to a sterile bottle and centrifuged at 15,900 × *g* for 10 min. The cell pellet was suspended in 200 ml of a solution containing 10% (vol/vol) glycerol and 5% (vol/vol) ethylene glycol. Aliquots of the cell suspension (2 ml) were distributed into cryogenic vials and immediately frozen and stored at −80°C.

### Shake flask cultivation.

Shake flask reader (SFR) vario systems (PreSens Precision Sensing GmbH, Regensburg, Germany) were used to monitor pH and dissolved oxygen throughout the growth of shake flask cultures. For shake flask culturing, a single-use 2-liter polycarbonate baffled flask equipped with precalibrated pH and dissolved oxygen sensors (PreSens Precision Sensing GmbH) containing 400 ml YDFM was inoculated from a thawed seed culture (initial OD_600_, 0.01). The culture was supplemented with 100 μg/ml ampicillin and various concentrations (0.5% to 2% [wt/vol]) of glucose, depending on the experiment. The culture was grown at 30°C with shaking at 275 rpm. Culture pH and dissolved oxygen were measured every 5 min using PreSens flask studio software. After 14 to 16 h of growth, 1 ml of cell culture was sampled every hour. The cell density at each time point was determined by measuring OD_600_ with a spectrophotometer. Each sample was then centrifuged at 13,000 × *g* for 75 s, and the cleared culture medium was stored at 4°C until further analyzed.

### Bioreactor cultivation.

Dasgip bioblock fermenters controlled by DASware software (Eppendorf, Hauppauge, NY) were used for all bioreactor cultivation experiments. Fermenters were inoculated from thawed seed cultures (initial OD_600_, 0.01). The pH was maintained at 5.65 using 28% (vol/vol) ammonium hydroxide and 10% (vol/vol) phosphoric acid. The cultures were grown at 30°C and agitated at an initial speed of 500 rpm. When the dissolved oxygen reached 30% air saturation, an agitation/gas flow/oxygen enrichment cascade was used to maintain this level of oxygenation. Batch cultures used 1-liter YDFM containing 2% glucose (wt/vol) as a carbon source, 100 μg/ml ampicillin, and 0.01% (vol/vol) Antifoam 204 (Sigma). Fed-batch cultures used 700 ml YDFM containing 2% (vol/vol) glucose or 2% (vol/vol) glycerol, 100 μg/ml ampicillin, and 0.01% (vol/vol) Antifoam 204. After 18 h of growth, fed-batch cultures were supplemented with a feed solution containing 60% (vol/vol) glucose or 60% (vol/vol) glycerol, 100 μg/ml ampicillin, and the following vitamins: calcium pantothenate (20 mg/liter), myoinositol (16 mg/liter), nicotinic acid (10 mg/liter), and pyroxodine (4 mg/liter). The feed solution was added at a rate of 5 ml/h for ∼18 to 24 h. Starting at 30 h of growth, 230 μl of a 50% (vol/vol) Antifoam 204/ethanol solution was added to the culture every 6 h. The culture was sampled (1-ml aliquots) throughout the experiment to permit monitoring of metabolite production, nutrient consumption, and recombinant protein production. For each sample, the cell density of the culture was measured (OD_600_), after which the samples were centrifuged at 13,000 × *g* for 75 s, and cleared spent culture medium was stored at 4°C until analyzed.

### Metabolite measurements.

The consumption of carbon source and nutrients and production of metabolic products were monitored using a Cedex Bio HT analyzer (Roche Diagnostics, Indianapolis, IN). The analyzer was equipped with reagent packs and was used as recommended by the manufacturer.

### Gaussia luciferase activity assay.

*Gaussia princeps* luciferase activity was assayed using the BioLux *Gaussia* luciferase assay kit (New England Biolabs). Cleared spent culture medium was diluted 1:20 in 1× phosphate-buffered saline (PBS). A 2-μl aliquot of this solution was transferred to a 96-well black-bottom microwell plate (BD Biosciences, Franklin Lakes, NJ) containing 50 μl 1× PBS. GLuc assay solution (50 μl/well) was added by an injector-equipped LB960 Centro luminometer (Berthold Technologies, Bad Wildbad, Germany) at low speed. After a delay time of 1 s, the light emission corresponding to the GLuc enzymatic activity was measured (0.5-s counting time) in relative light units (RLU).

### α-l-Fucosidase activity assay.

The activity of α-l-fucosidase was measured using a colorimetric assay with 2-chloro-nitrophenyl α-l-fucopyranoside (CNPF; Carbosynth Limited, Compton, UK) as a substrate. Spent culture medium (10 μl) was added to 100 μl of a 20-mM potassium acetate solution (pH 5.5) containing 2 mM CNPF. This mixture was incubated at 37°C for ∼15 h. The reaction was stopped by the addition of 50 μl of 1 M sodium carbonate, and the absorbance of released 2-chloro-4-nitrophenol was measured at 405 nm using an Ultrospec 2100 pro spectrophotometer (GE Healthcare).

### Enterokinase activity assay.

Enterokinase (EK_L_) activity was determined using the fluorogenic enteropeptidase substrate GDDDDK-β-naphthylamide (Bachem, Torrance, CA). Cleared spent culture medium (50 μl) from a K. lactis YCT1267 culture was added to an equal volume of EK_L_ assay buffer (0.02 M Tris-HCl [pH 8.0], containing 0.05 M NaCl, 0.002 M CaCl_2_, and 0.7 mM GDDDDK-β-naphthylamide). Fluorescence (excitation at 337 nm and emission at 420 nm) was measured at 25°C every 60 s for 15 to 20 min using a SpectraMax M5 fluorescence spectrophotometer in kinetic mode (Molecular Devices, San Jose, CA). To generate an EK_L_ standard activity curve, different concentrations of EK_L_ (e.g., 0.1 to 10 U, depending upon the desired range) were suspended in 1× EK_L_ assay buffer. An equal volume of YDFM was added, and the reaction mixture was incubated at 25°C for 20 min and measured as described above. The data were subsequently plotted.

### Statistical analysis.

For all data points, the averages of no less than three biological replicates are reported (see individual figure legends for *n*). Error bars represent the standard deviation from the mean for each value shown. *P* values were calculated using Student’s two-tailed heteroscedastic *t* test. The symbol “****” indicates that the *P* value was ≤0.0001.

## Supplementary Material

Supplemental file 1
