# Supplementary material for: A Novel Regulated Hybrid Promoter That Permits Autoinduction of Heterologous Protein Expression in Kluyveromyces lactis
Source: Appl Environ Microbiol. 2019 Jul 1;85(14):e00542-19. doi: 10.1128/AEM.00542-19 (PMC6606884; doi:10.1128/AEM.00542-19)
Supplement: Supplemental file 1 [file AEM.00542-19-s0001.pdf]

Supplemental Material

A novel regulated hybrid promoter that permits auto-induction of heterologous protein expression in *Kluyveromyces lactis*

Hassan Sakhtah[1*], Juliane Behler[1,2*], Alana Ali-Reynolds[1], Thomas B. Causey[1], Saulius Vainauskas[1‡] and Christopher H. Taron[1‡§]

[*]Authors contributed equally to this work

[‡]Authors share senior authorship

[1] New England Biolabs, 240 County Road, Ipswich, MA 01938, USA

[2]Genetics & Experimental Bioinformatics, University of Freiburg, Institute of Biology III,

Schänzlestraße 1, D-79104 Freiburg, Germany

[§]Corresponding author. Email: taron@neb.com

This document provides information (tables and figures) supplemental to the main text

Table S1. DNA primers used in this study.

Primer Description	[1]Name	[2]Sequence 5' - 3'
KlADH3 expression cassette assembly	ADH3_S1A	cgttgtaaaacgacggccagtgaattCAAACCCGTAGTGATGCAC
	ADH3_S1B	gagaatttcatCTTAATATTTCTTGTTTTAATTTGATGGG
	ADH3_S1C	gaaatattaagATGAAATTCTCTACTATATTAGCCG
	ADH3_S1H	ccatccgaaatCTATGGAGTCACCACATTTC
	ADH3_S1I	tgactccatagATTTCGGATGGGTGCTTG
	ADH3_S1J	ggatccccgggtaccgagctcgaattcATGTAGAAACTCCGATTTTACATC
KlGAP1 expression cassette assembly	GAP1_S1A	cgttgtaaaacgacggccagtgaattcACCTCGGAAGATTCCCAATTAC
	GAP1_S1B	gagaatttcatTGTGTAATATTCTTTTTTTTTACTTGAAACTG
	GAP1_S1C	aatattacacaATGAAATTCTCTACTATATTAGCCG
	GAP1_S1H	tcggtactgacCTATGGAGTCACCACATTTC
	GAP1_S1I	tgactccatagGTCAGTACCGACTGGGATC
	GAP1_S1J	ggatccccgggtaccgagctcgaattcAGTATGACATGTCCGGTG
KlGUT2 expression cassette assembly	GUT2_S1A	cgttgtaaaacgacggccagtgaattCCATCAACCCCAGACAAAAAC
	GUT2_S1B	gagaatttcatCGTTTGTAAACTGTGAGCG
	GUT2_S1C	gtttacaaacgATGAAATTCTCTACTATATTAGCCG
	GUT2_S1H	tcgttcagcagCTATGGAGTCACCACATTTC
	GUT2_S1I	tgactccatagCTGCTGAACGAAAAAATTAAGG
	GUT2_S1J	ggatccccgggtaccgagctcgaattcGTTTTGTTTTGCGCTGGTTTTG
KlICL1 expression cassette assembly	ICL1_S1A	cgttgtaaaacgacggccagtgaattcATCTAGATAATCGGGTATGATTAAC
	ICL1_S1B	gagaatttcatTATGTTGGGTTTGTATGTTTTG
	ICL1_S1C	aacccaacataATGAAATTCTCTACTATATTAGCCG
	ICL1_S1H	aaaggtaaaagCTATGGAGTCACCACATTTC
	ICL1_S1I	tgactccatagCTTTTACCTTTGTTGTCTTATGTG
	ICL1_S1J	ggatccccgggtaccgagctcgaattcGCAGATTAGGTGAGCTTAC
KlSTR3 expression cassette assembly	STR3_S1A	cgttgtaaaacgacggccagtgaattcTTAACTAAATCAAAAGTTGACTTAATC
	STR3_S1B	gagaatttcatGTTGGAAGTTTATTGGTTGG
	STR3_S1C	aaacttccaacATGAAATTCTCTACTATATTAGCCG
	STR3_S1H	ttatgaaggctCTATGGAGTCACCACATTTC
	STR3_S1I	tgactccatagAGCCTTCATAATATTACTGGAC
	STR3_S1J	ggatccccgggtaccgagctcgaattCTACCTTACTGGTGAAACTTG
KlLAC4 expression cassette assembly	LAC4_S1A	cgttgtaaaacgacggccagtgaattcGATCGACTCATAAAATAGTAACC
	LAC4_S1B	gagaatttcatTTTTTTCAAGCTTCTCGATG
	LAC4_S1C	gcttgaaaaaaATGAAATTCTCTACTATATTAGCCG
	LAC4_S1H	atctgttccttCTATGGAGTCACCACATTTC
	LAC4_S1I	tgactccatagAAGGAACAGATAGATAAAATTCCG
	LAC4_S1J	ggatccccgggtaccgagctcgaattCCGCGGAAATTTAGGAATTTTAAAC
Expression cassette assembly	S1D	aaggggcctgtTCTTTTCTCGAGATCATCC
	S1E	tcgagaaaagaACAGGCCCCTTTTCCTTTGTCG
	S1F	cacccggaaacAGCTTGCAAATTAAAGCCTTC
	S1G	atttgcaagctGTTTCCGGGTGTACAATATG
Promoter specific backbone amplification	S2K	ACAGGCCCCTTTTCCTTTGTCG
	S2L	TCTTTTCTCGAGATCATCCTTGTCAG
Gaussia luciferase	Gluc_S2M	gacaaggatgatctcgagaaaagaAAGCCCACCGAGAACAACGAAG
	Gluc_S2N	atcgacaaaggaaaaggggcctgtTTAGTCACCACCGGCCCCCTTG
Fucosidase	Fuc_S2M	gacaaggatgatctcgagaaaagaTCATCATCATCATCATCATTACC
	Fuc_S2N	atcgacaaaggaaaaggggcctgtTCAGATAGCAGATATTTGTGAATG
Enterokinase	EK_S2M	gacaaggatgatctcgagaaaagaATTGTTGGTGGTTCTGATTCT
	EK_S2N	atcgacaaaggaaaaggggcctgtCTAATGTAGAAAACTTTGTATC
Expression cassette amplification primers	S3O	TATTACGCCAGCTGGCGAAAG
	S3P	GTGAGCGGATAACAATTTCACACAGG
pUC19/ICL1 partial backbone for fusion	PICL1(-680)_rev	GGCTGAGGAACCAAATAGAGTC
	PICL1_amdS_fwd	TGTTTCCGGGTGTACAATATGG
P1000 promoter	PICL1_PGAP1_fwd	atttggttcctcagccACCTCGGAAGATTCCCAATTAC
	PICL1_PGAP1_rev	tgtacacccggaaacaAGCTTGCAAATTAAAGCCTTCG
P500 promoter	PGAP1-500fusion_fwd	gactctatttggttcctcagccTGGACAGGAAGAGAAAATC
	PGAP1-500fusion_rev	ctaatatagtagagaatttcatTGTGTAATATTCTTTTTTTTTACTTGAAAC
P450 promoter	PGAP1-450_fusion_fwd	gactctatttggttcctcagccACTTTCACCAGATCCCAAATG
P400 promoter	PGAP1-400_fusion_fwd	gactctatttggttcctcagccTACCATAACTTACCATTTCATCAC
P350 promoter	PGAP1-350_fusion_fwd	gactctatttggttcctcagccTCGACTGCTTTGCTTCATC
P220 promoter	PGAP1-220_fusion_fwd	gactctatttggttcctcagccTAATTTTGATATATAAAGGGTGGATC
P125 promoter	PGAP1-125_fusion_fwd	gactctatttggttcctcagccTTCTTATTAACCTTTTTTTTAAGTCAAAAC
P74 promoter	PGAP1-74_fusion_fwd	gactctatttggttcctcagccTCAAAGGTATTTCAAGTTATCATAC

[1]Primers correlate to specific expression vector cloning steps outlined in Figure S1. Primers with S1, S2 or S3 in their name pertain to cloning steps 1, 2 or 3, respectively. The letter immediately following denotes the exact location of the primer within that schematic.

For example, EK-S2M and EK-S2N that are used in Step 2 of the cloning strategy, anneal to locations M and N, and are used to amplify DNA encoding enterokinase. P### primers were used to prepare truncated PICL1-PGAP fusion promoters.

[2]Uppercase letters represent insert-specific DNA sequences; lowercase letters are sequences that overlap with the vector backbone or an adjacent DNA region.

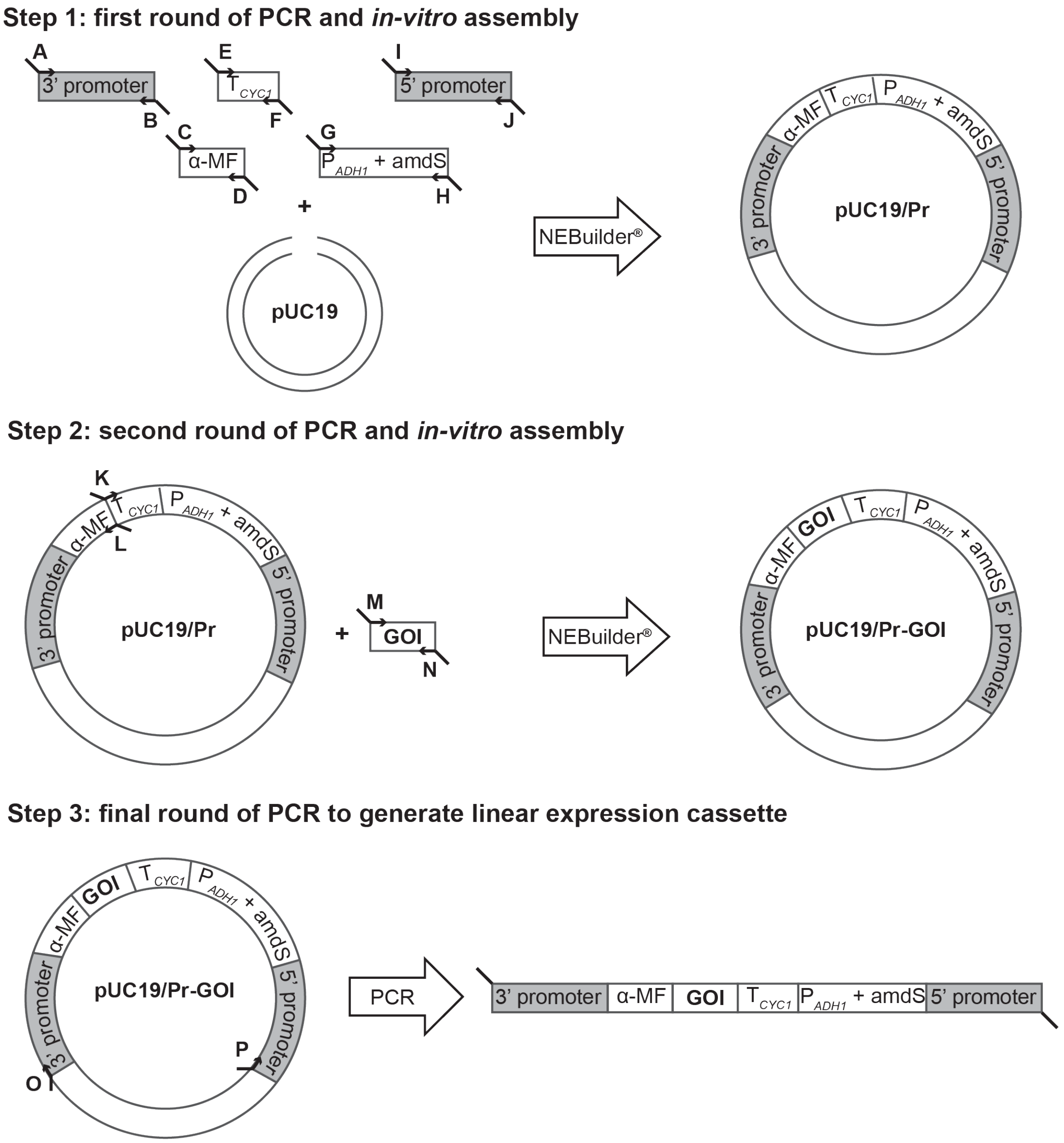

Figure S1. Assembly of *K. lactis* expression cassettes *in vitro*. (Step 1) Different promoters used in this study (3' Promoter) and their flanking upstream regions needed for homologous recombination (5' Promoter) were amplified from *K. lactis* GG779 genomic DNA. *Saccharomyces cerevisiae* YJM193 genomic DNA was used for the amplification of the *CYC1* transcription terminator sequence (T_{CYC1}). The pKLAC2 vector (NEB, Ipswich, MA) was used as template for amplifying the *Aspergillus nidulans* acetamidase expression cassette (P_{ADH1} + amdS) and *K. lactis* α-mating factor secretion leader sequence (α-MF). Primers were designed to create homologous overlaps between the different fragments (black arrows) and the letters (A to P) denoting each primer correspond to primer sequences presented in Table S1. The EcoRI digested vector pUC19 was used to assemble individual expression constructs. The fragments were assembled *in vitro* into EcoRI-linearized pUC19 plasmid DNA using NEBuilder® HiFi DNA Assembly Master Mix, resulting in a plasmid pUC19/Pr. (Step 2) DNA fragments from a reporter gene of interest (GOI) and from pUC19/Pr were amplified by PCR (both using primers with homologous overlaps) and assembled to create a new plasmid containing a complete integrative expression cassette. (Step 3) A linear integrative expression cassette was prepared by PCR using specific primers and the assembled plasmid from Step 2 as a DNA template. This linear cassette was used for integrative transformation of *K. lactis* cells.

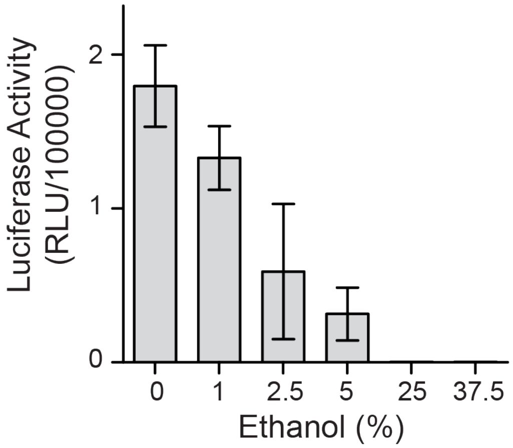

Figure S2. Sensitivity of *Gaussia princeps* luciferase to ethanol. *K. lactis* P_{350}-Gluc was grown in a fermenter in YDFM containing 2% glucose. After 41.5 h, 1 mL of culture was removed and centrifuged at 13000 x *g* for 1 min to pellet the cells. The cleared spent medium was diluted 1:20 in phosphate buffered saline (PBS). An aliquot of the dilution (2 μL) was added to PBS containing different quantities of ethanol, and Gluc activity was measured. Error bars represent standard deviation from the mean of three technical replicates.

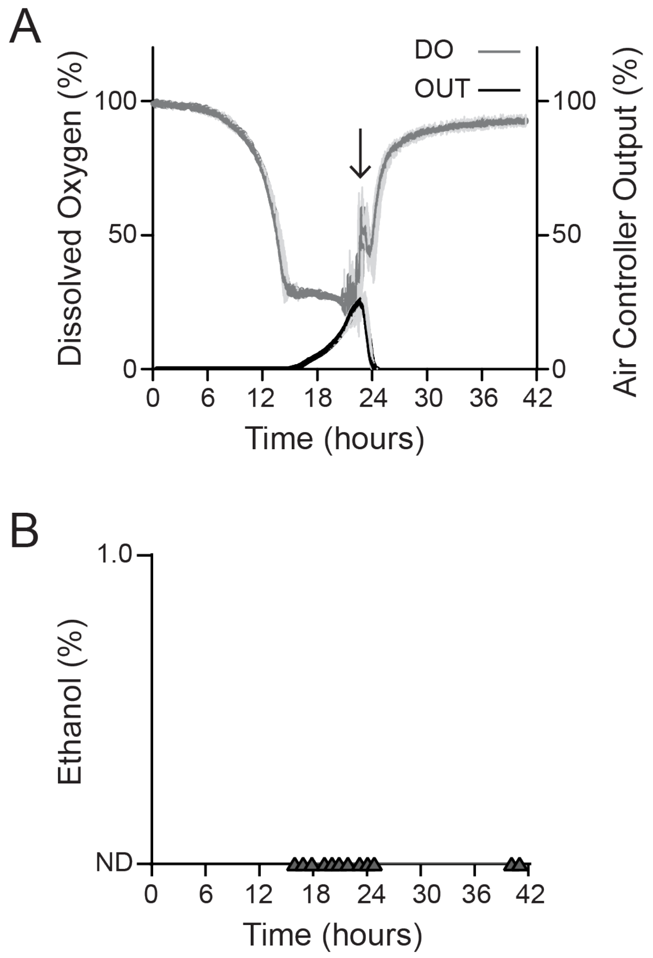

Figure S3. Additional bioprocessing measurements for *K. lactis* fermentations presented in Figure 5A-B of the main text. (A) Shown is average oxygen consumption (dark gray line), dissolved oxygen controller output (black line) relative to the time of culturing. The arrow indicates time at which glucose became growth-limiting to the cells. Light grey shading represents standard deviation from the mean of three replicate fermentations. (B) Shown is ethanol production relative to the time of culturing. ND represents the ethanol (<0.05%) detection limit of our instrumentation.

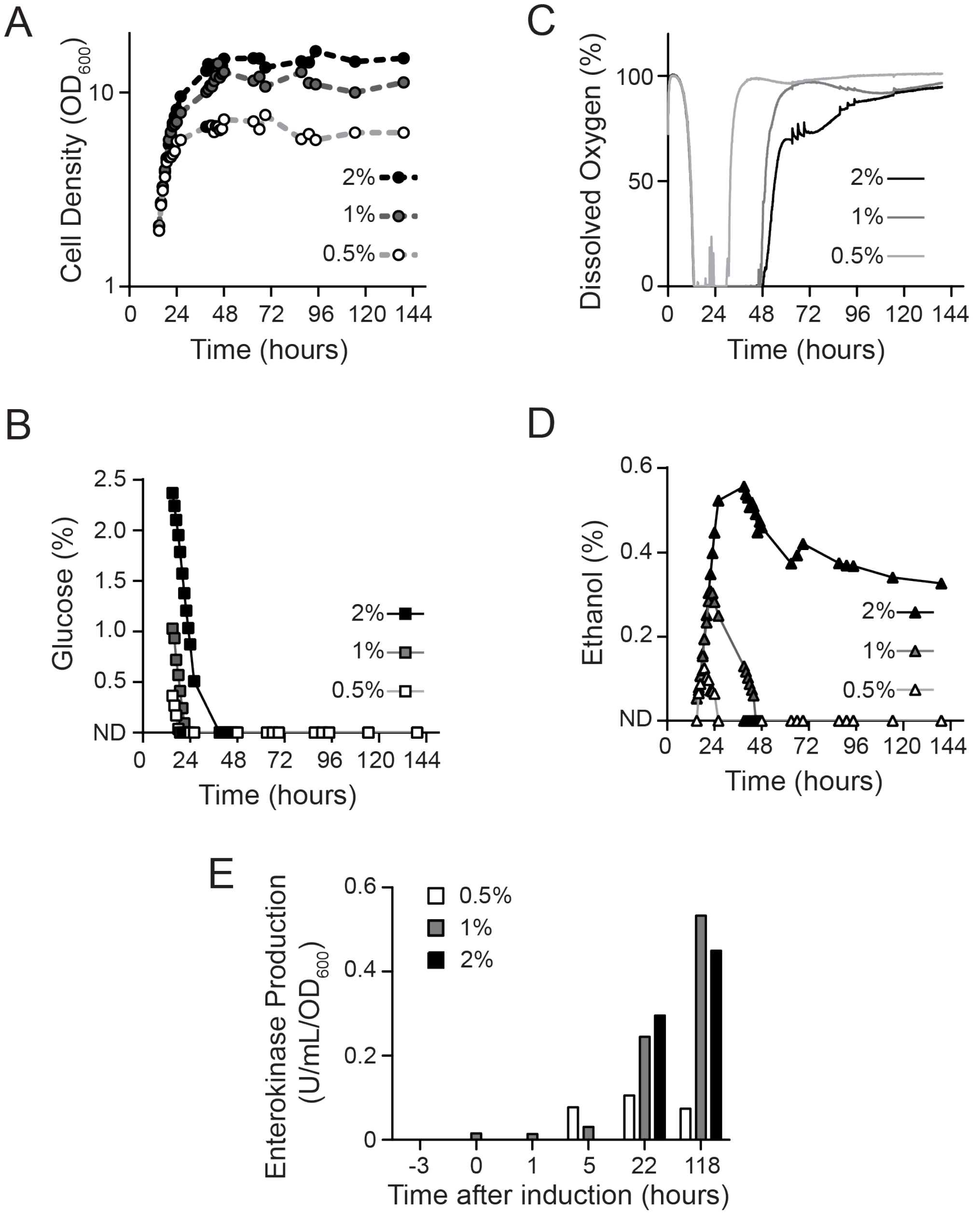

Figure S4. Shake flask production of enterokinase in growth medium having different glucose concentrations. Shown are (A) cell growth, (B) glucose consumption, (C) oxygen consumption, and (D) ethanol production, each relative to the time of culturing. (E) Shown is EK_L production relative to the time at which glucose became depleted and P_{350}-EK_L was induced (induction time) in each culture. Enterokinase production was calculated by normalizing EK_L activity (U/mL) to the cell density (OD_{600}) of each culture at each timepoint post-induction. In panels B and D, ND represents the glucose (<0.002%) and ethanol (<0.05%) detection limits of our instrumentation. In all panels, light gray lines, grey lines, and black lines represent cultures grown in 0.5%, 1% and 2% glucose, respectively.

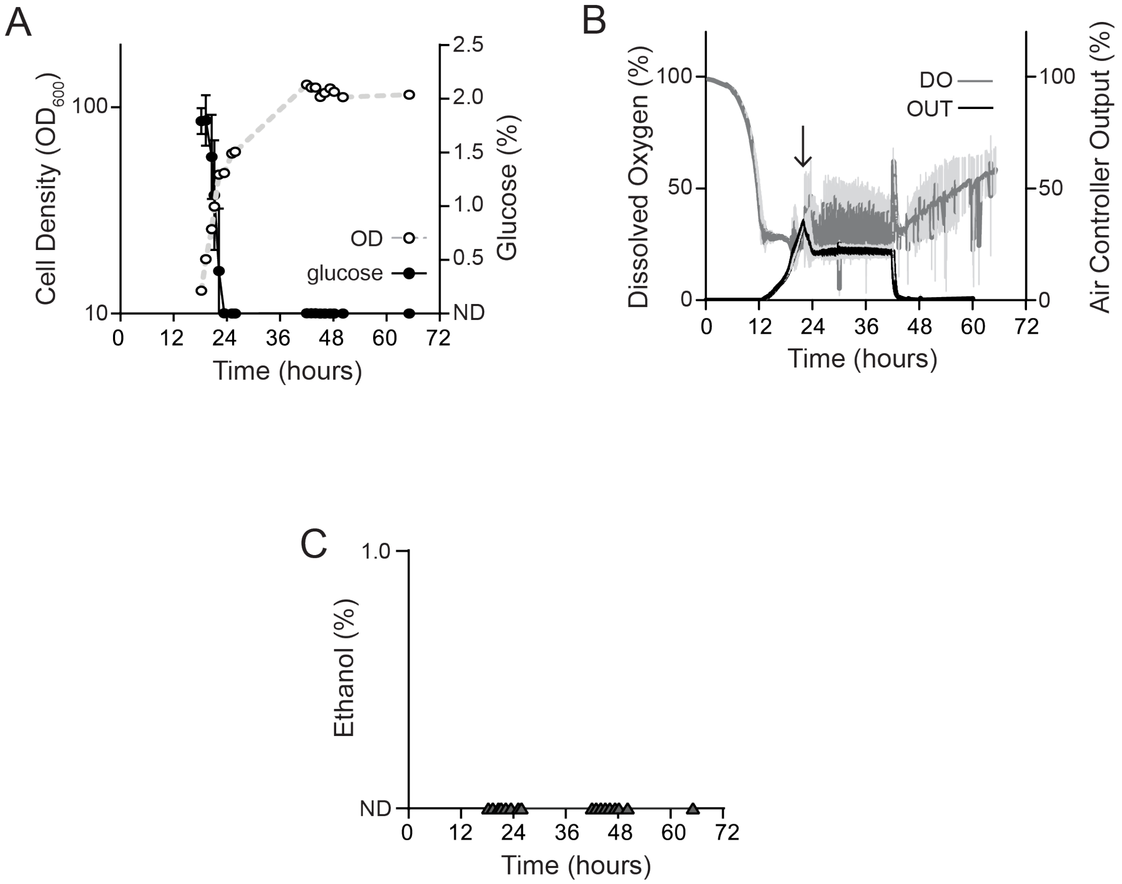

Figure S5: Additional bioprocessing measurements for *K. lactis* high cell density fermentations presented in Figure 5C of the main text. Shown are (A) glucose consumption, (B), oxygen consumption, and (C) ethanol production, each relative to the time of culturing. In panel B, dark gray lines represent average dissolved oxygen (DO) and the black line represents average oxygen controller output (OUT) of three replicate cultures. The arrow indicates time at which glucose became growth-limiting to the cells. In panels A and C, ND represents the glucose (<0.002%) and ethanol (<0.05%) detection limits of our instrumentation. In all panels, error bars or light gray shading represents standard deviation from the mean of three replicate fermentations.

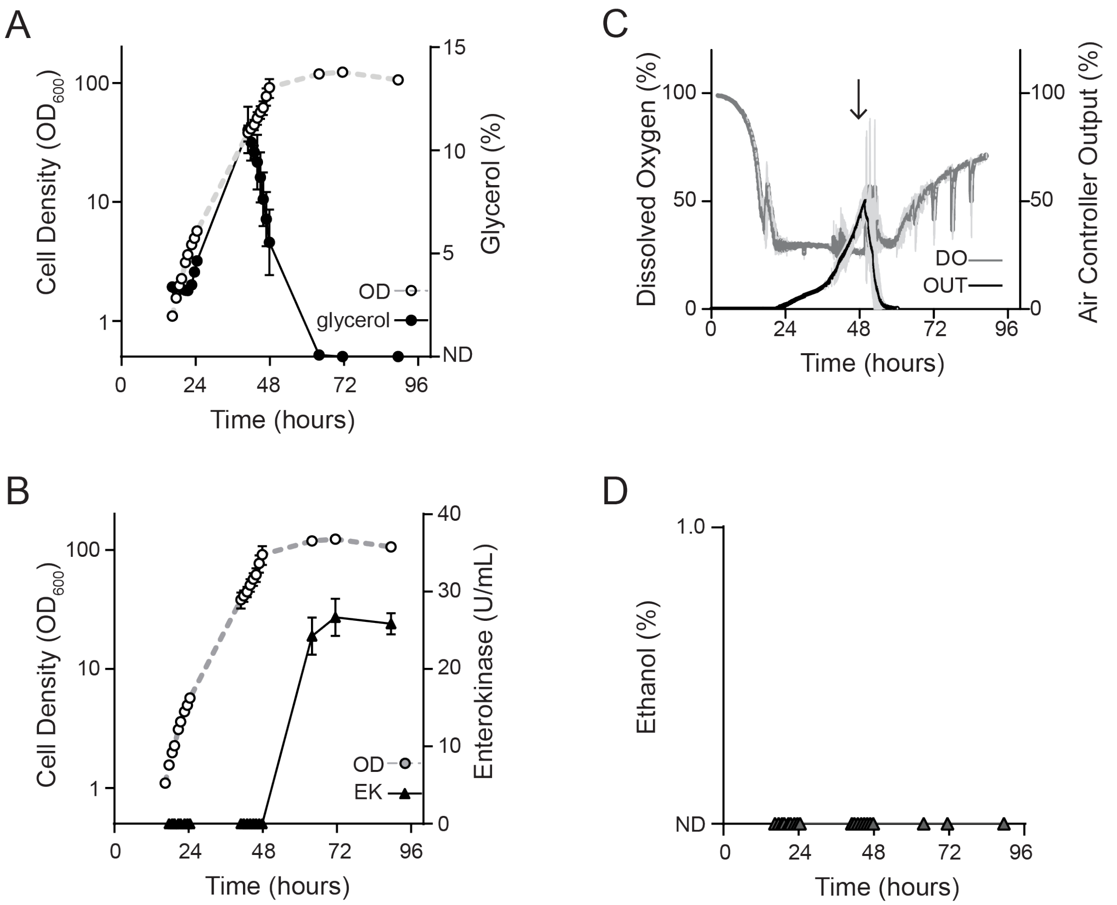

Figure S6. P_{350}-dependent production of enterokinase by *K. lactis* YCT1267 grown in high cell density fermentations using glycerol as a carbon source. Triplicate fermentations of *K. lactis* YCT1267 were performed in bioreactors. A 60% glycerol feed solution containing ampicillin and vitamins was added at a constant rate of 5 mL/h from 22.5 h (OD_{600} = ~4.3) to 41 h (OD_{600} = ~38.2) to achieve high cell density. Shown are (A) glycerol consumption, (B) EK_L activity, (C) oxygen consumption, and (D) ethanol production, each relative to the time of culturing. In panel B, dark gray lines represent average dissolved oxygen (DO) and the black line represents average oxygen controller output (OUT) of the three replicate cultures. The arrow indicates time at which glucose became growth-limiting to the cells. In panels A and D, ND represents the glycerol (<0.0009%) and ethanol (<0.05%) detection limits of our instrumentation. In all panels, error bars or light gray shading represents standard deviation from the mean of three replicate fermentations.
